# Impact of the COVID-19 pandemic on admissions of deceased to an institute of legal medicine in Germany

**DOI:** 10.1038/s41598-025-97117-w

**Published:** 2025-04-29

**Authors:** Kristina Allgoewer, Christiane Stark, Antonia Fitzek, Tobias Huter, Axel Heinemann, Benjamin Ondruschka

**Affiliations:** Institute of Legal Medicine, University Medical Center Hamburg-Eppendorf, Hamburg, Germany

**Keywords:** COVID-19 deaths, Pandemic, Germany, Autopsies, Post mortem examination, Legal medicine, Public health determinants, Diseases, Health care, Medical research

## Abstract

All over the world, the COVID-19 pandemic has impacted mortality beyond deaths that can be directly attributed to the viral disease. This study investigates the effects of the pandemic on admissions of deceased to a large institute of legal medicine and metropolitan morgue in Germany. Employing statistical models, the general time trend was separated from the effect of the pandemic in terms of place of death, autopsy category, age and sex. In addition, the pandemic’s impact on one of the structurally most important public health determinants, poverty, on numbers of admissions in different place of death categories was analyzed. We find that the pandemic has caused a significant increase in admissions of those who died at residential addresses, which appears to be primarily driven by SARS-CoV-2 negative cases, and those who died in retirement and care facilities, with a significant overrepresentation of SARS-CoV-2 positive cases. A high degree of poverty in a neighborhood does not impact the likelihood to be admitted to the institute in those two categories before or during the pandemic. For dead bodies found in public spaces, however, a poverty variable causes a significant increase in the number of admissions during the pre-pandemic phase. Interestingly, this effect is reversed during the pandemic period. The number of admissions without an autopsy being ordered or requested increased significantly during the pandemic. Further, the COVID-19 pandemic caused a significant increase in admissions in the senile population. Our results indicate that the reluctance of treating physicians to conduct house calls to certify a death has persisted throughout the pandemic and has caused a surge of admissions of deceased to the institute of legal medicine without any criminological indications or subsequent rise in forensic autopsy orders.

## Introduction

The COVID-19 pandemic has impacted mortality all over the world in various ways^1–4^. By October 2023, close to 7 million confirmed COVID-19 deaths had been counted worldwide^5^. However, for 2020 and 2021 alone, the World Health Organization (WHO) reported 14.83 million excess deaths, although just 36.5 percent are directly attributable to COVID-19^6^. While not all COVID-19 deaths could be correctly identified due to insufficient or missing testing, a simultaneous rise in non-COVID-19 deaths may partly be caused by an increase in non-natural scenarios such as substance abuse, suicides, and domestic violence due to mental health struggles during the pandemic and may therefore not be completely independent from the pandemic era^7–9^. In addition, delays in medical care and routine check-ups due to lockdowns and an overburdened health system may have led to preventable non-COVID-19 deaths^10–12^. On the other hand, the stay-at-home orders during the pandemic also led to a decrease in traffic deaths and fatalities caused by any other infectious diseases^13,14^.

While the impact of the pandemic on causes of death has been extensively covered^7–14^, less attention has been given to its effect on mortuary operations, including the admission of corpses, places of death, death certification and autopsy categories at forensic institutions. When a human body is found dead in Germany, an external post mortem examination needs to be performed by a physician without delay. While regulations regarding the certification of death differ across German states, the examination generally includes an ascertainment of death as well as an assessment of the cause and manner of death (natural versus non-natural or unclear). A non-natural or unclear death will trigger a police investigation to determine the likelihood of any third-party guilt. While the treating physician is evidently the most suitable person to perform the external post mortem for death certification due to her/his knowledge of the prior medical history of the deceased, the task may be considered undesirable due to challenging external conditions, inadequate training, controversial medical fee schedules and potential pressure from authorities as well as relatives to attest a natural death to avoid potentially burdensome investigations^15–17^.

In the northern German Federal State of Hamburg, one of three German city-states, all deceased for whom a natural death has not yet been attested by the certifying physician, are transported to the Institute of Legal Medicine at the University Medical Center Hamburg-Eppendorf for a second and detailed external examination by a forensic pathologist. A simultaneous police investigation determines whether the corpse may be released to a funeral home or needs to be further examined via autopsy and subsequent investigations due to suspicious circumstances and a non-negligible likelihood of third-party involvement in the death^18^. In addition, the institute serves as the morgue for all deaths occurring at the University Medical Center, meaning that all patients that pass away during hospitalization or outpatient treatment are transported to the Institute of Legal Medicine for specialized external post mortem examination. Further, forensic pathologists at the Institute of Legal Medicine perform clinical as well as private autopsies requested by the clinical colleagues or the family of the deceased as well as autopsies ordered by insurance institutions and forensic autopsies issued by prosecution authorities from neighboring states. In addition, forensic pathologists perform autopsies for research purposes as well as for the recovery of corneas and other (cardiovascular and musculoskeletal) tissues for post mortem tissue donation after gaining permission of the family of the deceased. Autopsies may also be ordered by public health authorities according to §25(4) of the German Infection Protection Act^19^. Historically, these autopsies are referred to as “administrative autopsies” in Germany.

During the COVID-19 pandemic, all corpses admitted to the Institute of Legal Medicine in Hamburg, were screened for SARS-CoV-2 RNA with nasopharyngeal swabs and quantitative reverse transcription PCR from March 3, 2020 until the end of 2022. Systematic autopsies were performed in more than 330 cases with proven SARS-CoV-2 infection, leading to crucial discoveries regarding the pathophysiology of the novel disease^20–23^. A study from the German COVID-19 autopsy registry showed that until October 2021, COVID-19 was the underlying cause of death in 86 percent of autopsy cases with SARS-CoV-2 infection. 249 out of 1129 documented autopsies in Germany were performed in Hamburg^24^.

To explore the impact of the COVID-19 pandemic on one of the largest institutes of legal (forensic) medicine and metropolitan morgues in Europe, we draw on a unique dataset of admissions of deceased showing information on demographics, places of deaths, and type of autopsy performed (if any) for the time span from 2018 to mid-2023, allowing us to examine developments caused by the COVID-19 pandemic compared to time frames without pandemic influences. Using this data, we employ statistical models that allow to distinguish between the pandemic scenario and a non-pandemic counterfactual in order to causally identify the effects of the pandemic.

## Results

### Pandemic effects on places of death

**Fig. 1:**
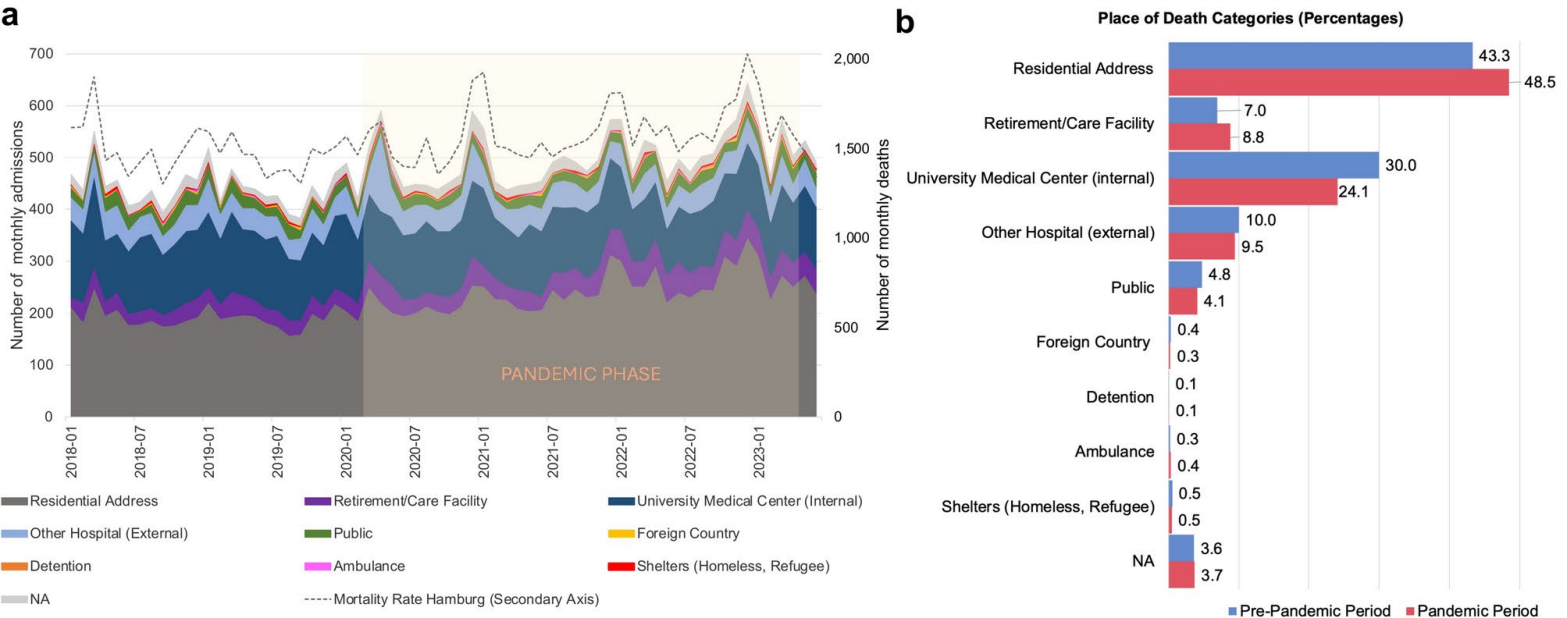
Admissions of deceased categorized by places of death from 2018 to mid-2023. (**a**) The monthly admissions to the Institute of Legal Medicine in Hamburg (left axis) are divided into 9 categories for places of death (see Data Collection). The mortality rate in the city-state of Hamburg (dashed line) is shown on the right axis. The pandemic phase according to the WHO definition (March 11, 2020 to May 5, 2023) is highlighted in a light-orange shade. (**b**) Percentages for each place of death category for admissions to the Institute of Legal Medicine are shown for the pre-pandemic phase (blue) and the pandemic phase (red). Missing values are marked as NA (not available).

The overall number of monthly admissions to the Institute of Legal Medicine in Hamburg has increased over the course of the COVID-19 pandemic, with four main peaks for the first wave starting in March 2020 as well as for each subsequent winter season (Fig. 1a). Analyzing absolute numbers, a bivariate Poisson regression shows a significant rise of admissions in three place of death categories: those of individuals who died in residential addresses, those who died in retirement and care facilities, and those who were transferred to the institute from other (non-university) hospitals for an autopsy. A significant decrease can be seen for admissions from the university medical center as well as for dead bodies found in public spaces (Supplementary Table S1). In percentage terms, however, the share of admissions from other hospitals slightly decreased during the pandemic period, while a distinct increase can be seen for deaths in residential addresses and retirement and care facilities (Fig. 1b and Supplementary Fig. S1). Hypergeometric testing confirms the significant overrepresentation of admissions from residential addresses and retirement and care facilities during the pandemic period and shows a significant underrepresentation of admissions from the university medical center as well as for dead bodies found in public spaces (Table 1). Repeating the analysis just for the subgroup of SARS-CoV-2 positive decedents, however, reveals a significant underrepresentation of admissions from residential addresses, while admissions from retirement and care facilities are even more frequently represented (Supplementary Table S2).

**Table 1:** Changes in the number of admissions in different place of death categories before and during the COVID-19 pandemic.

Place of death category	Status in pandemic	Fold change	P-value
Residential address	Over-represented***	1.039	< 0.001
Retirement/care facility	Over-represented***	1.081	< 0.001
University medical center (internal)	Under-represented***	0.918	< 0.001
Other hospital (external)	Under-represented	0.981	0.089
Public	Under-represented**	0.940	0.003
Foreign country	Under-represented	0.946	0.288
Detention	Over-represented	1.104	0.277
Ambulance	Over-represented	1.137	0.055
Shelters (Homeless, Refugee)	Under-represented	0.936	0.177
NA	Over-represented	1.028	0.126

Parameters to calculate the p-value for under- or over-representation are based on the cumulative distribution function of the hypergeometric distribution. Fold change as compared to expected value. Missing values are marked as NA (not available). Significant results in bold; *** p ≤ 0.001, ** p ≤ 0.01.

**Fig. 2:**
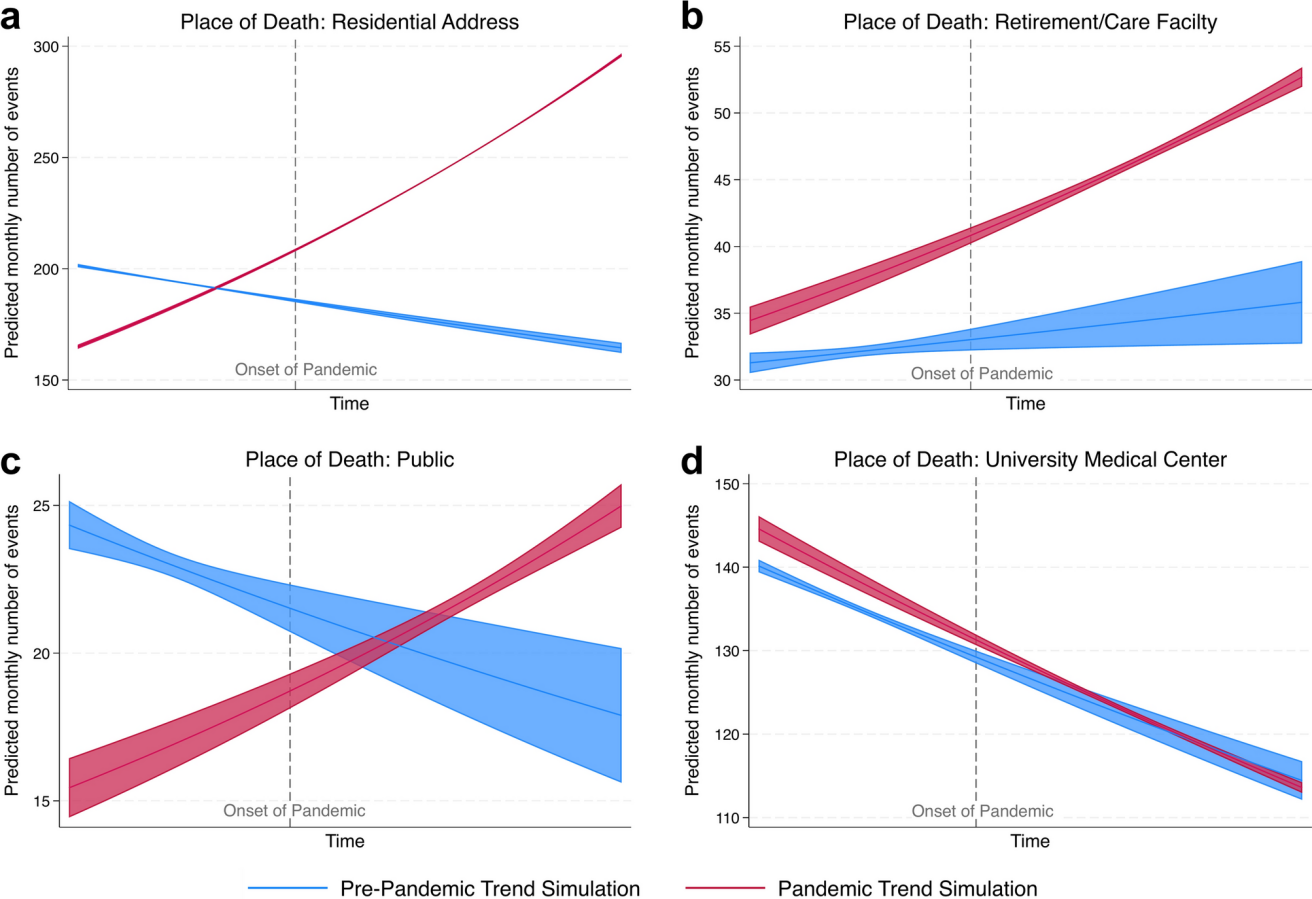
Admissions by place of death: How did the pandemic impact the trend? Predicted number of monthly admissions to the Institute of Legal Medicine categorized by place of death if the pandemic had never occurred (blue) or if the pandemic had already existed prior to the actual onset (red), analyzed in a Poisson regression using the number of monthly admissions in each category as the dependent variable and the respective month, the pandemic dummy variable and the multiplicative interaction term of both as independent variables. (**a**) Significant impact on category “residential address”: Increasing the value of the time variable by one standard deviation from its mean increases the number of predicted monthly admissions by 18.6% under the pandemic scenario, while we would have expected a decrease by 5.8% under a non-pandemic scenario. (**b**) Significant impact on category „retirement/care facility”: Increasing the value of the time variable by one standard deviation from its mean increases the number of predicted monthly admissions by 13.2% under the pandemic scenario, while we would have expected an increase by only 4.0% under a non-pandemic scenario. (**c**) Significant impact on category “public”: Increasing the value of the time variable by one standard deviation from its mean increases the number of predicted monthly admissions by 15.1% under the pandemic scenario, while we would have expected a decrease by 8.6% under a non-pandemic scenario. (**d**) For the category “University Medical Center”, a slightly significant positive impact of the pandemic on admissions can only be detected in the early phase of the pandemic, with trends converging over the course of the observation period.

To be able to determine whether these developments were caused by a general time trend rather than the pandemic itself, an interaction term was added to the Poisson regression. The resulting visualizations (Fig. 2) simulate how admissions for the four place of deaths categories in question would have developed if the pandemic had never happened (blue) and if the pandemic situation had prevailed for the whole observation period (red). The pandemic caused a significant increase in admissions from residential addresses (Fig. 2a). Without a pandemic situation, a slight decrease in admissions would have been expected in this category (for effect sizes see figure legend). Further, a significant increase in admissions from residential addresses and retirement and care facilities was caused by the pandemic (Fig. 2b). In contrast to the category of residential addresses, however, this only leads to a strengthening of the trend instead of a trend reversal. In the place of death category “public” the onset of the pandemic initially led to a sudden and distinct decline of admissions and over the whole pandemic period the percentage of admissions relative to the other categories decreased. However, since the number of admissions started to rise in the later phases of the pandemic (after lockdowns ended), a positive effect of the pandemic (with trend reversal; Fig. 2c) can be observed. For admissions of deceased patients from the university medical center, a slight significant increase can be determined in the initial stages of the pandemic (Fig. 2d). Over time, trends align for both the pandemic and non-pandemic scenarios.

**Fig. 3:**
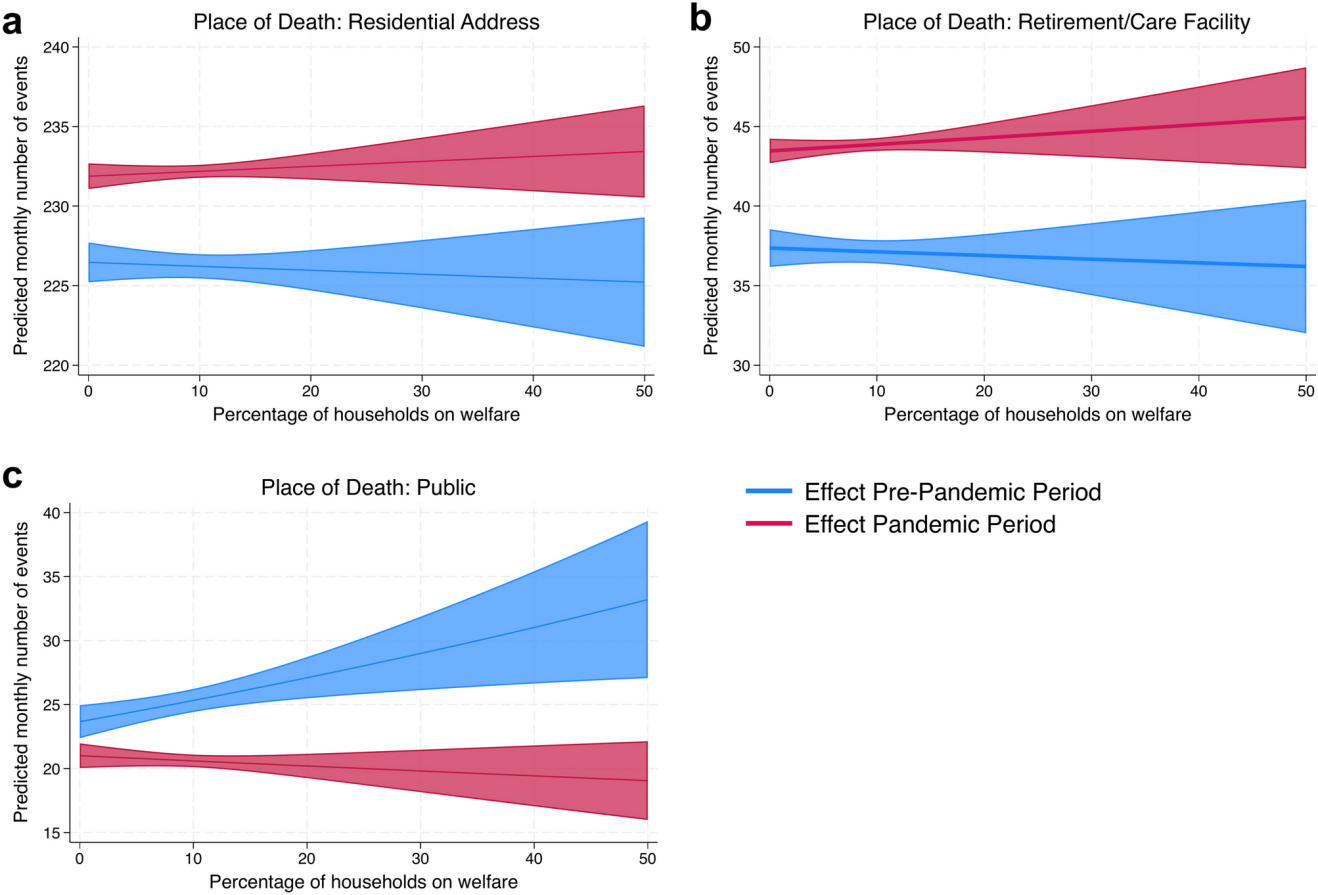
Admissions by place of death: What impact do structural variables such as poverty have? Predicted number of monthly admissions to the Institute of Legal Medicine categorized by place of death if the pandemic had never occurred (blue) or if the pandemic had already existed prior to the actual onset (red), analyzed in a multivariate Poisson regression using the number of monthly admissions in each category as the dependent variable and a poverty variable for the respective neighborhood, the pandemic dummy variable and the multiplicative interaction term of both as well as further structural variables (see Methods) as independent variables. We don’t see any effect of the poverty variable on the number of admissions in the categories “residential address” (**a**) and “retirement/care facility” (**b**). In the category “public” (**c**) a higher degree of poverty in a neighborhood leads to an increase in the expected number of admissions in a pre-pandemic situation, but not in a pandemic situation.

Since health outcomes often depend on structural variables such as poverty^25^, we were interested in similar effects on the place of death categories. The results of a multivariate Poisson analysis using the percentage of households receiving public welfare benefits per neighborhood as a poverty variable are shown in Fig. 3 and Table 2. As there is no variation in the structural variables of the place of death for patients who died at the university medical center, this analysis was only performed for the three remaining categories (residential address, retirement and care facilities, and public spaces). The analysis shows no significant changes in the effect of the poverty variable on admissions from residential addresses or retirement and care facilities (Fig. 3a,b), even though the pandemic itself led to a significant increase in the number of admissions in both categories. For dead bodies found in public spaces (Fig. 3c) a higher percentage of welfare recipients in the respective neighborhood causes a significant increase in the number of admissions during the pre-pandemic phase. Interestingly, this effect disappears—and is even slightly reversed—during the pandemic phase. The number of physicians per capita and the percentage of residents older than 64 years per neighborhood don’t have a significant impact on the number of admissions in any category. The overall mortality in Hamburg only has a significant impact on admissions from residential addresses and retirement/care facilities, but not on those found in public spaces.

**Table 2:** Admissions by place of death: What impact do structural variables such as poverty have?

Variables	(A) Residential address	(B) Retirement/care facility	(C) Public
	Coefficients (standard errors in parentheses)		
Poverty (percentage of households receiving welfare benefits)	−0.0001 (0.0002)	−0.0006 (0.00140)	0.0068** (0.002)
Dummy variable for pandemic	0.024*** (0.003)	0.151*** (0.0184)	−0.119** (0.039)
Pandemic * Poverty (interaction term)	0.0002 (0.0003)	0.0016 (0.0015)	−0.0087** (0.0028)
Physicians per capita	−0.026 (0.250)	2.51 (1.219)	1.72 (1.95)
Percentage of residents > 64 years	−0.0001 (0.0001)	−0.0005 (0.0008)	−0.0017 (0.0016)
Time trend (months)	0.004*** (0.00006)	0.004*** (0.0003)	0.0048*** (0.0007)
Mortality in Hamburg (Monthly number of deaths)	0.0007*** (3.67e−06)	0.0006*** (0.00002)	−0.00008 (0.00005)
Constant	1.209*** (0.0406)	−0.187 (0.222)	−0.160 (0.496)
Likelihood ratio Chi^2^	88,556.34***	3897.29***	72.49***
Observations	13,354	2394	1026

Poisson regression results for monthly number of admissions in three categories of places of death (dependent variable) with an interaction term for the poverty variable and the pandemic dummy variable while controlling for structural variables for the neighborhood in which the dead body was found as well as the time trend and the monthly number of deaths in Hamburg. A higher degree of poverty in a neighborhood has a significant and positive impact on the number of admissions of dead bodies found in public places. However, this effect was reversed during the pandemic (see interaction term). Significant results in bold; *** p ≤ 0.001, ** p ≤ 0.01.

### Pandemic effects on autopsy categories

**Fig. 4:**
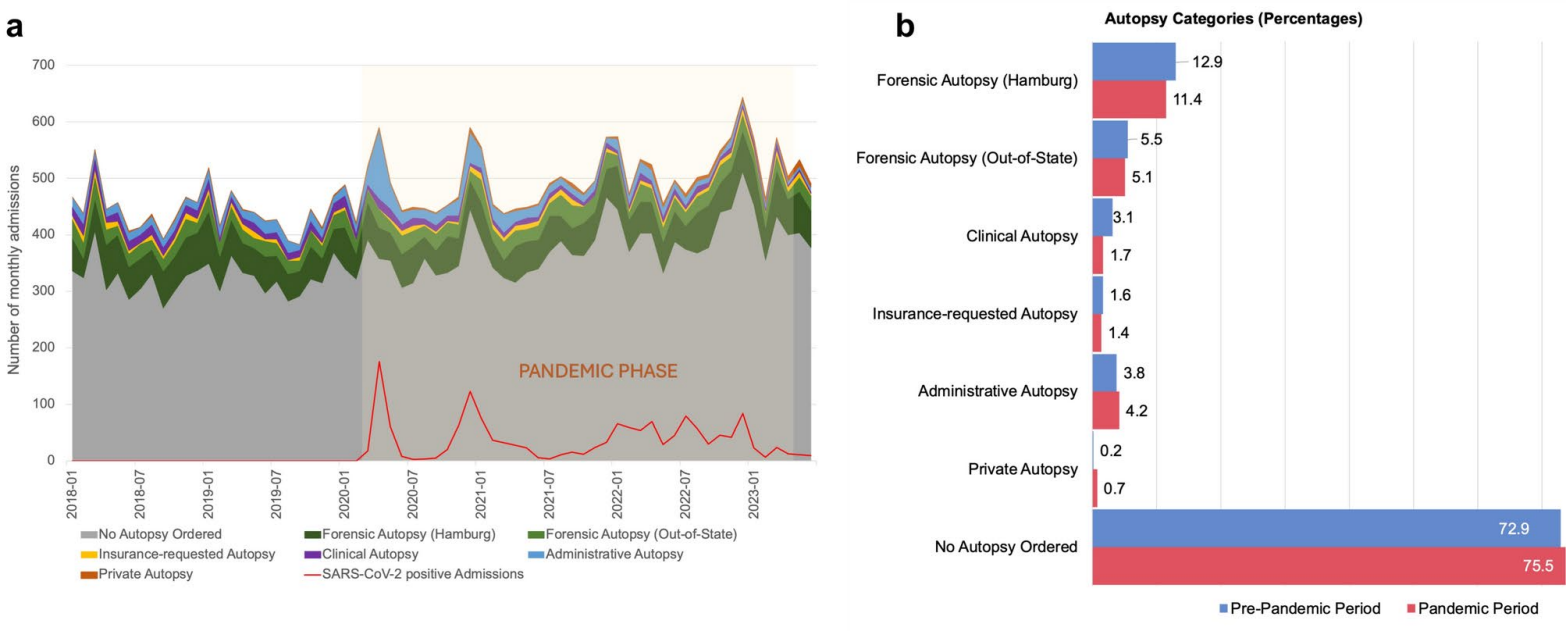
Admissions of deceased by autopsy category from 2018 to mid-2023. (**a**) The monthly admissions to the Institute of Legal Medicine in Hamburg are divided into 6 autopsy categories as well as those admissions for which no autopsy is ordered and performed. The red line shows the number of monthly admissions with a confirmed post mortem SARS-CoV-2 infection. The pandemic phase according to the WHO definition (March 11, 2020 to May 5, 2023) is highlighted in a light-orange shade. (**b**) Percentages for each autopsy category are shown for the pre-pandemic phase (blue) and the pandemic phase (red).

The majority of bodies admitted to the Institute of Legal Medicine in Hamburg will be released to funeral homes after an external examination without an autopsy being ordered or requested (Fig. 4a). Even though the overall number of admissions increased during the observation period, the percentage of all autopsies declined from 27.1 percent in the pre-pandemic period to 24.6 percent in the pandemic period (Fig. 4b).

In absolute numbers, the bivariate Poisson regression shows significant developments across all categories, with a significant rise in forensic autopsies ordered by out-of-state authorities, administrative autopsies, private autopsies and admissions without autopsies being performed, as well as a significant decline in forensic autopsies ordered by Hamburg authorities, clinical autopsies and autopsies requested by insurances (Supplementary Table S3). Percentage-wise the only categories showing an increase are administrative autopsies, private autopsies and admissions without autopsies being performed, all of which show a significant over-representation during the pandemic in a hypergeometric test. Meanwhile, forensic autopsies ordered by Hamburg authorities, clinical autopsies, and insurance-requested autopsies are significantly under-represented in the pandemic phase (Table 3). Repeating the analysis for the subgroup of SARS-CoV-2 positive cases reveals a significant over-representation of administrative autopsies alone (Supplementary Table S4).

**Table 3:** Changes in the number of admissions in different autopsy categories before and during the COVID-19 pandemic.

Autopsy category	Status in pandemic	Fold change	P-value
Forensic autopsy (Hamburg)	Under-represented***	1.055	< 0.001
Forensic autopsy (Out-of-state)	Under-represented	1.028	0.085
Clinical autopsy	Under-represented***	1.272	< 0.001
Insurance-requested autopsy	Under-represented*	1.068	0.048
Administrative autopsy	Over-represented**	1.065	0.002
Private autopsy	Over-represented***	1.300	< 0.001
No autopsy ordered	Over-represented***	1.012	< 0.001

Parameters to calculate the p-value for under- or over-representation are based on the cumulative distribution function of the hypergeometric distribution. Fold change as compared to expected value. Significant results in bold; *** p ≤ 0.001, ** p ≤ 0.01, * p ≤ 0.05.

Again, an interaction term was added to the Poisson regression to be able to determine whether these developments were merely caused by a general time trend rather than the pandemic itself for these six significant categories (Supplementary Fig. S2). The onset of the pandemic initially caused a sharp decline in forensic autopsies ordered by Hamburg authorities. However, since orders for forensic autopsies picked up again during later stages of the pandemic, there is an overall positive effect. For clinical autopsies, the pandemic caused the strengthening of a downward trend that would also have occurred if the pandemic had never happened. While insurance-requested autopsies decreased during the pandemic, a scenario without a pandemic would have predicted an even greater decline. The category of administrative autopsies, which includes autopsies for research purposes ordered by public health authorities, saw a massive increase during the first wave of the pandemic. During later stages of the pandemic, however, the number of administrative autopsies declines. The pandemic has a significant impact on the number of private autopsies: An increase can be observed under the pandemic scenario, while a decline would have been expected if the pandemic had not occurred. For admissions without an autopsy being ordered, the pandemic caused a significant strengthening of an upward trend: More bodies were admitted to the institute without receiving an autopsy than would have been expected in a non-pandemic situation.

### Pandemic effects on age and sex categories

**Fig. 5:**
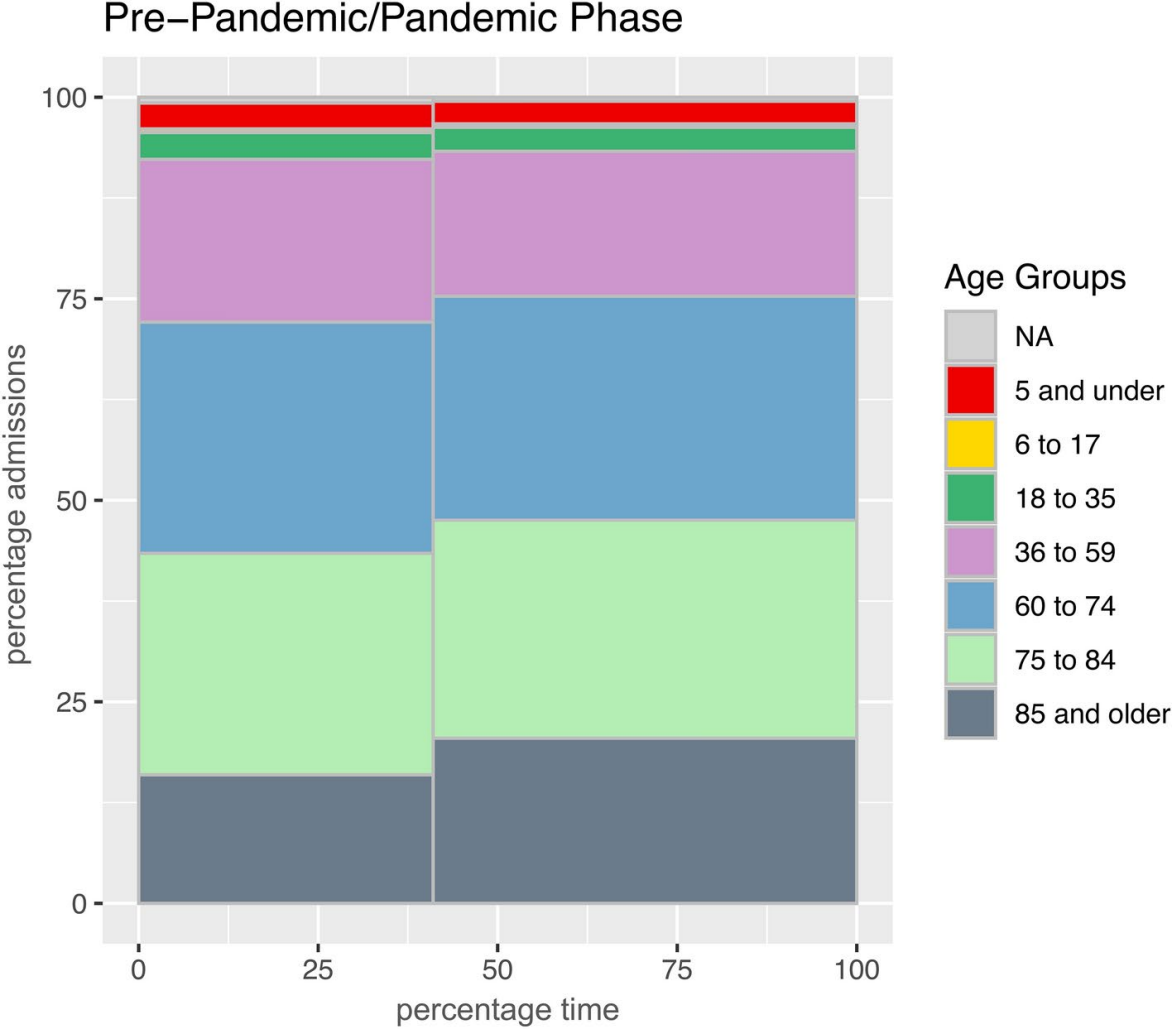
Percentages of admissions to the Institute of Legal Medicine divided into age categories prior to the pandemic (left column) as well as during the COVID-19 pandemic period (right column). Admissions in the 85 + age category were significantly overrepresented in the pandemic phase.

**Fig. 6:**
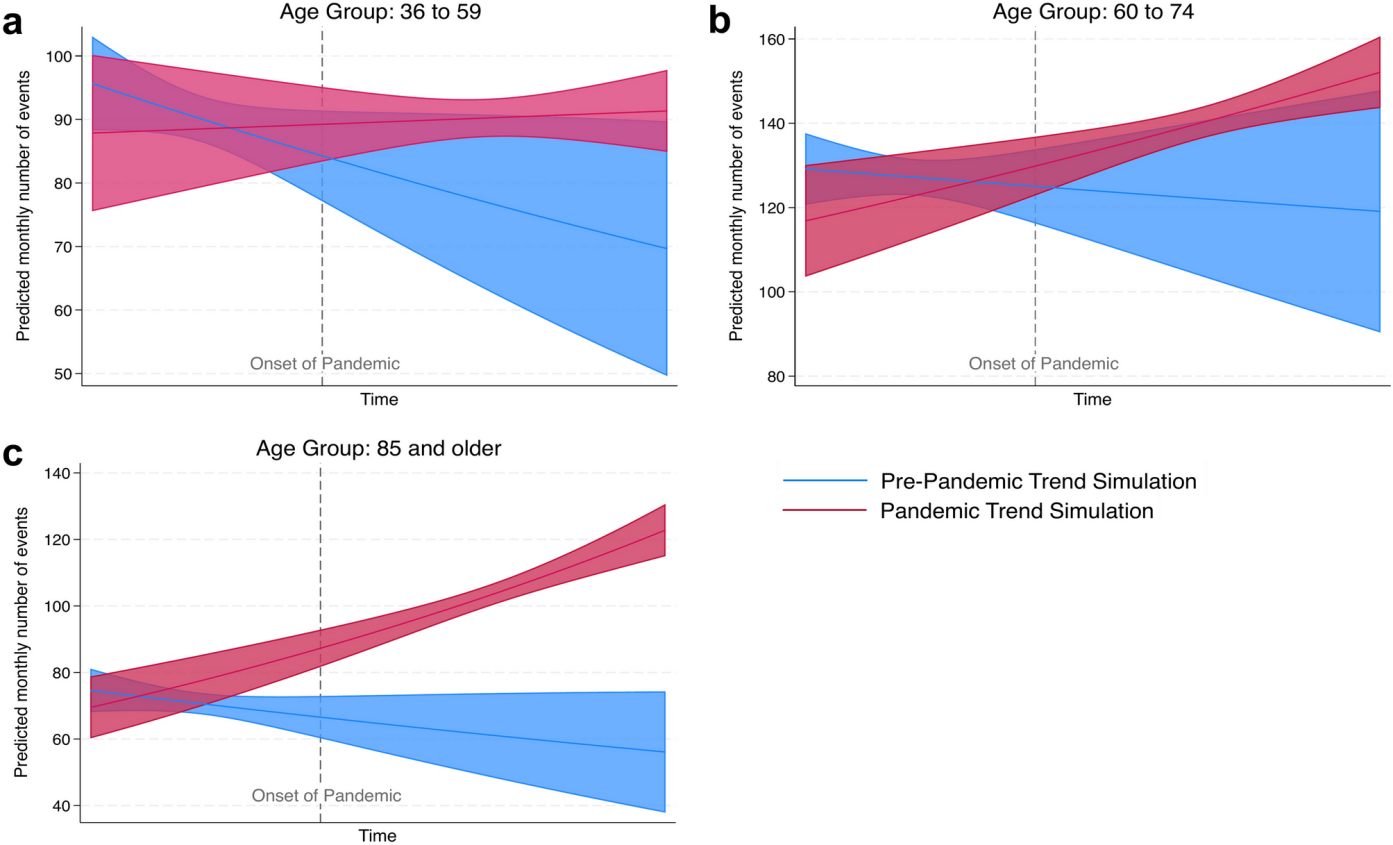
Admissions by age category: How did the pandemic impact the trend? Predicted number of monthly admissions to the Institute of Legal Medicine categorized by age if the pandemic had never occurred (blue) and if the pandemic had already existed prior to the actual onset (red), analyzed in a Poisson regression using the number of monthly admissions in each category as the dependent variable and the respective month, the pandemic dummy variable and the multiplicative interaction term of both as independent variables. For admissions in the age categories 36 to 59 years (**a**) and 60 to 74 years (**b**), a significant impact of the pandemic cannot be distinguished from a general time trend. However, the pandemic had a significant impact on admissions in the age category 85 years and older (**c**). Increasing the value of the time variable by one standard deviation from its mean increases the number of predicted monthly admissions by 17.6% under the pandemic scenario, while we would have expected a decrease by 8.0% under a non-pandemic scenario.

Admissions to the institute were split into seven age categories: five and younger, six to 17, 18 to 35, 36 to 59, 60 to 74, 75 to 84 as well as 85 and older. In absolute numbers, a rise in all age categories can be seen in a bivariate Poisson analysis, with a significant increase for the three highest age categories (Supplementary Table S5). Percentagewise, however, only admissions in the 85 + category were significantly over-represented in the pandemic phase, while admissions in the categories 36 to 59 and 60 to 74 were significantly under-represented (Fig. 5, Table 4). Yet, when distinguishing the impact of the pandemic from a general time trend through a Poisson analysis with an interaction term, the 85 + group remained the only group with a significant result: Increasing the value of the time variable by one standard deviation from its mean increases the number of predicted monthly admissions of deceased people 85 years and older by 17.6 percent under the pandemic scenario, while a decrease by 8.0 percent would have been expected if the pandemic had never occurred (Fig. 6). For the subgroup of SARS-CoV-2 positive admissions, a significant overrepresentation can be seen for the two highest age groups (75 years and older; Supplementary Table S4). Categorizing admissions to the Institute of Legal Medicine by sex (male, female, undetermined) shows a male majority, but no significant difference between the pre-pandemic and the pandemic period (61.3 vs 59.9 percent; Supplementary Fig. S3). For SARS-CoV-2 positive cases, the proportion of male admissions was 58.3 percent.

**Table 4:** Changes in the number of admissions in different age of death categories before and during the COVID-19 pandemic.

Age category	Direction	Fold change	P-value
5 and under	Under-represented	0.962	0.077
6 to 17	Under-represented	0.972	0.372
18 to 35	Under-represented	0.964	0.085
36 to 59	Under-represented***	0.954	< 0.001
60 to 74	Under-represented*	0.986	0.024
75 to 84	Under-represented	0.994	0.218
85 and older	Over-represented***	1.090	< 0.001
NA	Under-represented	0.939	0.192

Parameters to calculate the p-value for under- or over-representation are based on the cumulative distribution function of the hypergeometric distribution. Missing values are marked as NA (not available). Fold change as compared to expected value. Significant results in bold; *** p ≤ 0.001, * p ≤ 0.05.

## Discussion

The given data show that the COVID-19 pandemic has caused a significant increase in admissions to the Hamburg Institute of Legal Medicine of those who died at residential addresses, which appears to be largely driven by SARS-CoV-2 negative cases, as well in admissions from retirement and care facilities, with a significant overrepresentation of SARS-CoV-2 positive cases. However, this increase is not accompanied by a rise in forensic autopsies ordered by state authorities—on the contrary, the percentage of admissions not being followed by any type of autopsy order has significantly increased throughout the pandemic.

These results indicate that since the start of the pandemic, the treating physicians (general practitioners) are less likely available or interested to perform the external post mortem examination at the place of death after a dead body has been found. An emergency physician, who is in almost all cases unfamiliar with the medical history of the deceased, will most commonly not be able to certify a natural death. Instead, after the determination of death, they are authorized to issue a “preliminary certificate of death” without a detailed post mortem examination, which states the cause of death as “unclear”. This results in the need for a secondary external post mortem examination by another physician in most of the German federal states^26,27^ and specifically by a forensic pathologist in Hamburg^28–30^.

At the Institute of Legal Medicine and its central morgue in the Hamburg catchment area, this development has led to a persisting increase in admissions of bodies without any ‘typical’ indication for a non-natural death. Other German cities and regions have installed a designated panel of doctors with considerable experience in post mortems (although not necessarily being forensic experts) for routine examinations at the place of death. The ‘Hamburg way’, however, establishes twice-daily serial post mortem examinations in a specialized environment in the department’s morgue, while death scene investigations by forensic staff are only requested in suspicious or homicide cases by the Hamburg state authorities.

During the initial lockdown phase, primary care services in and around Hamburg were gravely affected. The highest reduction of services by general practitioners was reported in the category “house calls and calls at nursing homes”, for which the number was almost cut in half^31^. In a nation-wide survey, 28.5% of general practitioners reported fewer visits to severely ill and dying patients^32^. Whether the custom of performing house calls has ever resumed fully after the pandemic, is currently questionable. For primary care visits, an already declining trend prior to the pandemic may have been exacerbated by the implementation of tools for telemedicine^33,34^. When it comes to post mortem examinations, experience shows that the reluctance of general practitioners to perform home visits may have persisted in the aftermath of the COVID-19 pandemic, likely caused by a habituation effect.

Mortality during the COVID-19 pandemic has been impacted by socio-economic disparities in Germany and world-wide^35,36^. Looking at the results for the poverty variable in this analysis, however, it is interesting to note that regarding the place of death, the pandemic seems to have had virtually no negative effect on poorer neighborhoods of Hamburg. The increase in admissions from residential addresses and retirement and care facilities during the pandemic seems to affect both low- and high-income neighborhoods alike and is independent from structural factors such as the number of physicians per capita.

Per consensus in Hamburg, all people discovered deceased in public spaces are delivered to the Institute of Legal Medicine for a forensic external post mortem examination. As expected during a period that included reduced public space activity, the overall number of admissions in this place of death category decreased significantly during the pandemic period. While there is no detailed, systematic nationwide documentation of places of deaths in Germany, a decline of deaths in public spaces and an increase in deaths at home during the pandemic have been reported in other countries, consistent with our findings^37,38^. Independently from this trend, our results indicate a development that may be deemed positive from a public health perspective: While during the pre-pandemic phase the number of people who are found dead in public increased with the poverty level of a neighborhood, this effect vanished during the pandemic and was even slightly reversed. Put differently, instead of increasing disparities, the pandemic may have operated as an equalizer in this regard, potentially due to changes in social dynamics: In England, for instance, high-risk social mixing during the pandemic was found to be associated with lower financial hardship^39^.

Aside from the category “no autopsy ordered”, the autopsy categories “private autopsy”, i.e., autopsies requested by family members of the deceased, and “administrative autopsy” were over-represented during the COVID-19 pandemic in our data (compared to an expected value, which is based on the counts across all categories during the pandemic phase and the development of the respective category during the whole observation period), while all other categories were under-represented compared to expected values. In Hamburg, administrative autopsies include those ordered by health authorities to investigate the pathophysiology of the novel COVID-19 disease based on the German Infection Protection Act, which was modified during the first months of the pandemic to facilitate the process of autopsy orders^19^. To the best of our knowledge, Hamburg was the only city using this type of autopsy order in a frequent and regular manner in 2020 and 2021. The increase in administrative autopsies during the pandemic appears to have been primarily driven by SARS-CoV-2 positive decedents from other hospitals, who were admitted to the institute specifically for research and public health purposes.

A rise in the demand for private autopsies, on the other hand, seems to be primarily influenced by SARS-CoV-2 negative cases and is consistent with media reports in the United States, which speculate that more family-requested autopsies are being performed by private autopsy companies due to public officials and medical professionals being overwhelmed with the pandemic death toll and therefore being less likely to request an autopsy themselves. Further, the extensive media coverage on autopsies and their benefits during the pandemic may have contributed to the new popularity among those who are looking for answers regarding the deaths of family members^40^.

The dominance of males in the presented dataset is typical for a population containing a strong forensic sub-cohort. As “(high) risk seekers”, men are more likely than women to die prematurely and in non-natural scenarios such as accidents, suicides, and homicides all over the world^41,42^. During the pandemic phase, the percentage of males dropped slightly below sixty percent (change not significant). While male COVID-19 patients are at a higher risk of fatal outcomes^43–45^, the additional admissions in the male sex category might have been compensated by a decrease in male-prone non-natural death scenarios such as road traffic accidents during lockdown^14^ as well as by the greater life expectancy of women in Germany^46^. Since COVID-19 mortality is higher at older ages, a higher absolute number of women would have been affected. Our findings suggest that age may be a more significant risk factor than sex in determining fatal COVID-19 outcomes. The significant impact of the pandemic on admissions in the senile age category is consistent with mortality data of COVID-19 patients^20,47^ but may also demonstrate the increasing share of admissions of those who require a post mortem examination at the Institute of Legal Medicine after dying from natural causes at home and in retirement and care facilities.

## Limitations of this study

The analysis presented in this study is mainly based on routine data collected by the admissions clerk in a frequently extremely busy setting from the sole legal medicine institution in Hamburg, Germany. While some of the data base entries were either missing information or included implausible information, a majority could be re-evaluated and amended retrospectively. Although not permanently confronted with new or emerging infectious diseases within the typical service sector of forensic medicine and science, the institute intentionally decided to investigate as many SARS-CoV-2 positive death cases as possible from the very beginning of the pandemic. While we have explored to what extent those who tested positive for SARS-CoV-2 in our institution ultimately died from COVID-19 or from other causes in a previous study, this information is only available for a subset of our data and was therefore not included in this analysis^24^.

For several of the evaluated categories, the trend for admissions changes drastically over the course of the pandemic, leading to artifacts in the visualized simulations. While the analysis might benefit from a breakdown into several pandemic phases like the virus waves, a subdivision is prohibited by the sparsity of data, particularly in the low-frequency categories. We are also aware that the evaluation of the post-pandemic phase as a third time period likely would be able to harmonize some of the developments discovered and allow for a clearer comparison of the long-term effects and potential shifts in trends. However, given the evolving nature of the post-pandemic context and the need for a more comprehensive dataset, the collection of sufficient post-pandemic data would cause a considerable delay and should therefore be evaluated in future studies. Future research could additionally focus on a longitudinal approach to better understand how these changes stabilize or evolve over time, ultimately contributing to a more nuanced understanding of the lasting versus temporarily impacts of the pandemic on mortuary management and forensic institutions.

## Conclusion

The COVID-19 pandemic and the particular death certification regulations in Hamburg, Germany have caused a surge of admissions of deceased to the Institute of Legal Medicine without any criminological indications or subsequent rise in forensic autopsy orders. Lockdowns and the implementation of telemedicine seem to have exacerbated a decline in primary care home visits by general practitioners, which extend to post mortem examinations at the place of death, and which are independent from the socio-economic status of a neighborhood and even persist in the aftermath of the pandemic.

## Material and methods

### Data collection

For each body admitted to the Institute of Legal Medicine, a new data record is created in a password protected SQL database by the admissions clerk, containing personal data including age and sex, information on the residential address and the address of the place of death as well as a free-text field for commentary on special circumstances. The place of death is categorized by the admissions clerk into three groups: death at a residential address, death at the associated University Medical Center, or death at an external hospital. For this analysis of admissions from January 1, 2018 to June 30, 2023, the first category was fully re-evaluated and cases were subdivided into another six categories based on the address of the place of death and the information in the commentary: deaths that occurred in public spaces (such as parks, streets, train stations etc.), deaths in retirement and care facilities, deaths in homeless or refugee shelters (which often share the same street address), deaths in police custody or imprisonment (detention), deaths in ambulances, and deaths that occurred in foreign countries (with bodies arriving at the Hamburg port or airport). If none of these new categories applied, the place of death remained classified as “residential address”. In cases where an autopsy was performed, the suitable autopsy category (forensic, clinical, administrative, private, insurance-requested) was recorded by the prosector. The dataset also contains information on positive SARS-CoV-2 diagnoses in post mortem nasopharyngeal qPCR tests among the deceased since March 20, 2020.

### Statistical methods

#### Hypergeometric testing

Parameters to calculate the p-value for under- or over-representation of admissions in different categories (place of death, autopsy, age) were based on the cumulative distribution function (CDF) of the hypergeometric distribution using the HYPGEOM.DIST() function in Excel (Version 16.78.3; Microsoft, Redmond, WA, USA). The fold change shows the number of admissions in the respective category during the pandemic phase compared to an expected value, based on the counts across all categories during the pandemic phase, the counts in the respective category over the whole observation period, and the counts across all categories over the whole observation period.

#### Bivariate Poisson regression

Poisson regressions between a pandemic dummy variable and monthly numbers of admissions in different categories for place of death, autopsy categories and age groups were calculated in Stata 18.0 (StataCorp, College Station, TX, USA) using the poisson command. The pandemic dummy was coded as 1 for dates of admission from March 2020 to April 2023 and as 0 for dates from January 2018 to February 2020. The definition of this timeframe is based on the declaration by the World Health Organization, which declared COVID-19 a pandemic on March 11, 2020, and ended the status of Public Health Emergency of International Concern on May 5, 2023^48^. The two post-pandemic months in the dataset (May and June 2023) were omitted from the analysis due to the short time frame of this phase and the ensuing scarcity of data but are included in selected figures for visualization.

#### Poisson regression with interaction term

To investigate how the time trend is impacted by the pandemic, an interaction term between the month variable and the pandemic dummy variable was added to the poisson command in an additional analysis for the place of death and autopsy categories. The inclusion of an interaction term between the time trend and the pandemic variable serves to causally identify the impact of the pandemic on the outcome variable. It is assumed that the trend that is present in the data before the pandemic would have continued. If we see a significant change in the outcome quantity of interest due to the pandemic, this can be interpreted as brought about by the pandemic. The technique is inspired by difference-in-difference specifications frequently used in econometrics^49^. Interactions were visualized with the Stata command “margins” which calculates marginal effects at defined levels of the interacting variables and creates an interaction plot showing the marginal effects of covariates and their modification by the interacted variable together with the 95 percent confidence bands. To control for the number of monthly admissions to the Institute of Legal Medicine, this number was added as a covariate to the regressions for the different autopsy categories.

#### Poisson regression with covariates

Thirdly, we examined how the influence of a poverty variable on monthly counts in different place of death categories is changed by the pandemic in a multivariate Poisson analysis by adding a multiplicative interaction term between the poverty variable and the pandemic dummy variable to the poisson command. Structural data for Hamburg was retrieved from “neighborhood profiles” provided by the Federal Office of Statistics for Hamburg and Schleswig–Holstein for free (www.statistik-nord.de, data as of October 10, 2023). Zip codes from places of death were matched to 99 neighborhoods according to official records retrieved from the city website (https://www.hamburg.de/postleitzahlen/; data as of July 1, 2023). As poverty variable, we used the percentage of households with public welfare recipients (“Hartz4”) in a neighborhood. To avoid potential endogeneity problems, we used the percentage of households relying on welfare as of 2019. Further, we controlled for the number of physicians per capita and the percentage of people older than 64 years in the respective neighborhoods (data: years 2018 to 2022, with 2022 data being used for both 2022 and 2023 without factor) as well as for the time trend and the mortality rate. Official monthly mortality data for Hamburg was retrieved from the website of the German Federal Office of Statistics (http://www.destatis.de, data as of July 31, 2023).

#### Visualization

Stacked area plots were generated in Excel after the aggregation of count data in R 4.2.2 (open source; R Foundation for Statistical Computing, Vienna, Austria) using the dplyr package. Mosaic plots were generated in R using the packages ggplot2, reshape2, plyr and RColorBrewer. Line plots simulating the trend calculated from the coefficients of the Poisson analysis with interaction effects were generated in Stata 18.0.

### Ethics declaration

The given study solely involves the analysis of data and does not include any experiments on humans or animals. All described procedures were conducted in accordance with national law, and in compliance with the Declaration of Helsinki of 1975 (in its current, revised version). Institutional review board approval from the independent ethics committee of the Hamburg Chamber of Physicians was obtained (reference number 2020–10,353-BO-ff) and informed consent was obtained from the legal guardians or next of kin of the deceased for autopsies. The study was carried out in accordance with the guidelines of the Central Ethics Committee of the German Medical Association.

## Supplementary Information

Supplementary Information.

## Data Availability

The authors do not have permission to share data on individual cases for legal reasons. Aggregated data can be made available upon reasonable request via the corresponding author.
