# Supplementary material for: Impact of the COVID-19 pandemic on admissions of deceased to an institute of legal medicine in Germany
Source: Sci Rep. 2025 Apr 29;15:15115. doi: 10.1038/s41598-025-97117-w (PMC12041269; doi:10.1038/s41598-025-97117-w)
Supplement: Supplementary file 1 — Supplementary Information. [file 41598_2025_97117_MOESM1_ESM.pdf]

Supplementary Information

Impact of the COVID-19 pandemic on admissions of deceased to an institute of legal medicine in Germany

Authors: Kristina Allgoewer[1*], Christiane Stark[1], Antonia Fitzek[1], Tobias Huter[1], Axel Heinemann[1], Benjamin Ondruschka[1]

[1]*Institute of Legal Medicine, University Medical Center Hamburg-Eppendorf, Hamburg, Germany*

[*]*E-mail: k.allgoewer-martin@uke.de (corresponding author)*

Supplementary Figures

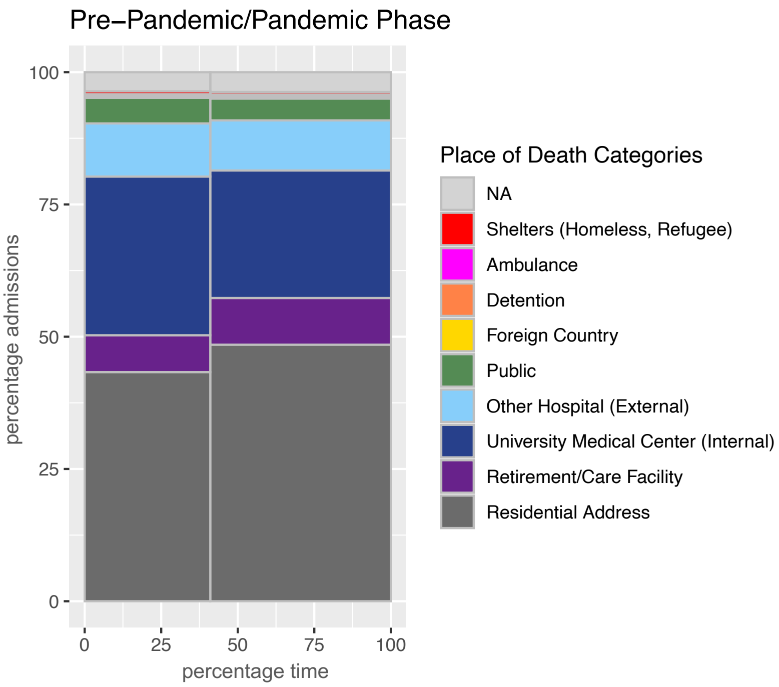

Supplementary Figure S1: Percentages of admissions to the Institute of Legal Medicine categorized by place of death prior to the pandemic (left column) as well as during the COVID-19 pandemic period (right column). Missing values are marked as NA (not available).

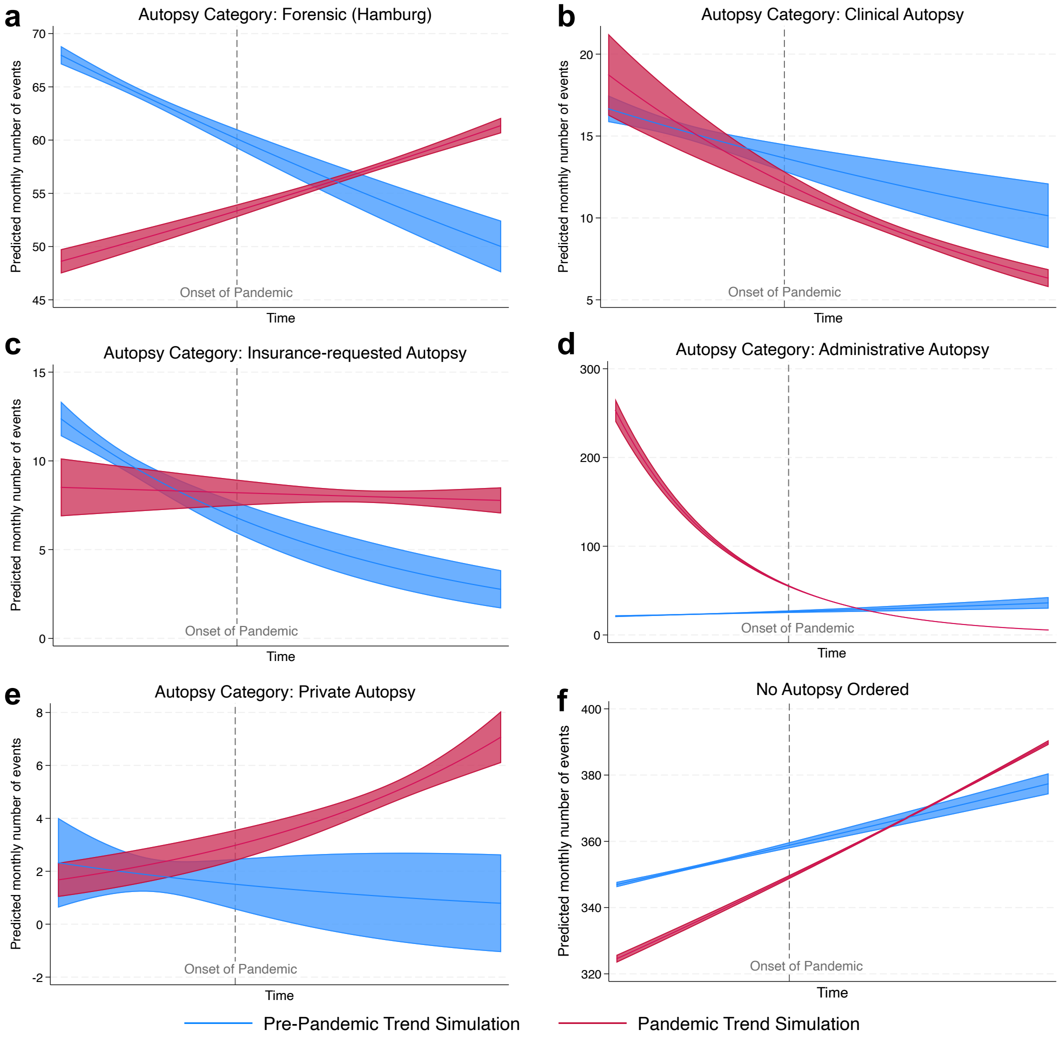

Supplementary Figure S2: Admissions by autopsy category: How did the pandemic impact the trend? Predicted number of monthly admissions to the Institute of Legal Medicine categorized by type of autopsy if the pandemic had never occurred (blue) or if the pandemic had already existed prior to the actual onset (red), analyzed in a Poisson regression using the number of monthly admissions in each category as the dependent variable and the respective month, the pandemic dummy variable and the multiplicative interaction term of both as independent variables and controlling for the overall number of monthly admissions. **a)** Significant impact on category “forensic autopsy (Hamburg)”: Increasing the value of the time variable by one standard deviation from its mean increases the number of predicted monthly admissions by 7.1% under the pandemic scenario, while we would have expected a decrease by 8.6% under a non-pandemic scenario. **b)** Significant impact on category “clinical autopsy”: Increasing the value of the time variable by one standard deviation from its mean decreases the number of predicted monthly admissions by 27.2% under the pandemic scenario, while we would have expected a decrease by only 13.6% under a non-pandemic scenario. **c)** Significant impact on category “insurance-requested autopsy”: Increasing the value of the time variable by one standard deviation from its mean decreases the number of predicted monthly admissions by 2.6% under the pandemic scenario, while we would have expected a decrease by 35.5% under a non-pandemic scenario. **d)** Significant impact on category “administrative autopsy”: Increasing the value of the time variable by one standard deviation from its mean decreases the number of predicted monthly admissions by 67.2% under the pandemic scenario, while we would have expected an increase by 17.0% under a non-pandemic scenario. **e)** Significant impact on category “private autopsy”: Increasing the value of the time variable by one standard deviation from its mean increases the number of predicted monthly admissions by 52.4% under the pandemic scenario, while we would have expected a decrease by 27.0% under a non-pandemic scenario. **f)** Significant impact on category “no autopsy ordered”: Increasing the value of the time variable by one standard deviation from its mean increases the number of predicted monthly admissions by 5.5% under the pandemic scenario, while we would have expected an increase by 2.5% under a non-pandemic scenario.

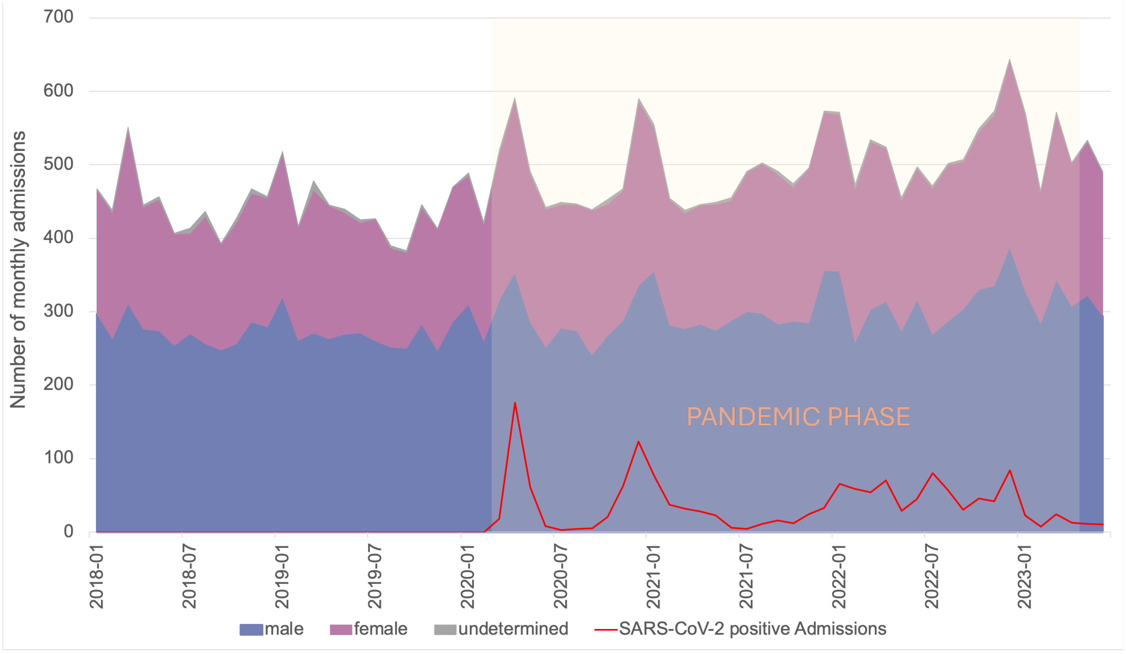

Supplementary Figure S3: Admissions of deceased categorized by sex from 2018 to mid-2023. The monthly admissions to the Institute of Legal Medicine in Hamburg categorized by sex (male, female, undetermined). The red line shows the number of monthly admissions with a confirmed SARS-CoV-2 infection. The pandemic phase according to the WHO definition (March 11, 2020 to May 5, 2023) is highlighted in a light-orange shade.

Supplemental Tables

Place of Death Category	Coefficient (z-value in parentheses)
Residential Address	**0.253*** (208.97)**
Retirement/Care Facility	**0.370*** (51.58)**
University Medical Center (internal)	**-0.093*** (-47.22)**
Other Hospital (external)	**0.200*** (1.84)**
Public	**-0.056*** (-4.81)**
Foreign Country	0.139 (1.10)
Detention	0.299 (1.06)
Ambulance	0.103 (0.77)
Shelters (Homeless, Refugee)	-0.035 (-0.39)
NA	**0.229*** (15.73)**

Supplementary Table S1: Changes in the place of death categories before and during the COVID-19 pandemic. Note: Bivariate Poisson regressions between pandemic dummy variable and monthly numbers of admissions in different categories for places of death (absolute numbers). Pandemic dummy is coded as 1 if month is from March 2020 to April 2023, 0 before March 2020. The two months after the pandemic (May and June 2023) are set to missing. Missing values are marked as NA (not available). Significant results in bold; *** p ≤ 0.001, ** p ≤ 0.01, * p ≤ 0.05. Constant terms not shown for clarity.

Place of Death Category	Status in COVID-19 confirmed	Fold Change	P-value
Residential Address	**under-represented*** **	0.677	< 0.001
Retirement/Care Facility	**over-represented*** **	2.041	< 0.001
University Medical Center (internal)	**under-represented*** **	0.853	< 0.001
Other Hospital (external)	**over-represented*** **	2.318	< 0.001
Public	**under-represented*** **	0.342	< 0.001
Foreign Country	**over-represented* **	1.898	0.048
Detention	over-represented	1.148	0.526
Ambulance	over-represented	1.252	0.346
Shelters (Homeless, Refugee)	under-represented	0.642	0.204
NA	over-represented	1.120	0.193

Supplementary Table S2: Number of admissions in different place of death categories for corpses with confirmed SARS-CoV-2 infection compared to all admissions. Note: Parameters to calculate the p-value for under- or over-representation are based on the cumulative distribution function of the hypergeometric distribution. Fold change as compared to expectations based on all data during the observation period. Missing values are marked as NA (not available). Significant results in bold; *** p ≤ 0.001, *p ≤ 0.05.

Autopsy Category	Coefficient (z-value in parentheses)
Forensic Autopsy (Hamburg Authorities)	**0.010* (-2.52)**
Forensic Autopsy (Out-of-State Authorities)	**0.060*** (6.08)**
Clinical Autopsy	**-0.390* (-17.31)**
Autopsy Requested by Insurance	**-0.087** (-2.72)**
Administrative Autopsy	**0.837*** (65.35)**
Private Autopsy	**0.890*** (5.66)**
No Autopsy Ordered	**0.170 *** (230.67)**

Supplementary Table S3: Changes in autopsy categories before and during the COVID-19 pandemic. Note: Bivariate Poisson regressions between pandemic dummy variable and monthly numbers of admissions in different autopsy categories (absolute numbers). Pandemic dummy is coded as 1 if month is from March 2020 to April 2023, 0 before March 2020. The two months after the pandemic (May and June 2023) are set to missing. Significant results in bold; *** p ≤ 0.001, ** p ≤ 0.01, * p ≤ 0.05. Constant terms not shown for clarity.

Autopsy Category	Status in COVID-19 confirmed	Fold Change	P-value
Forensic Autopsy (Hamburg)	**under-represented***	0.872	0.029
Forensic Autopsy (Out-of-State)	**under-represented*****	0.498	< 0.001
Clinical Autopsy	**under-represented*****	0.390	< 0.001
Insurance-requested Autopsy	under-represented	0.885	0.333
Administrative Autopsy	**over-represented*****	5.707	< 0.001
Private Autopsy	under-represented	0.817	0.372
No Autopsy Ordered	**under-represented*****	0.829	< 0.001

Supplementary Table S4: Number of admissions in different autopsy categories for corpses with confirmed SARS-CoV-2 infection compared to all admissions. Note: Parameters to calculate the p-value for under- or over-representation are based on the cumulative distribution function of the hypergeometric distribution. Fold change as compared to expectations based on all data during the observation period. Missing values are marked as NA (not available). Significant results in bold; *** p ≤ 0.001, * p ≤ 0.05.

Age Category	Coefficient (z-value in parentheses)
5 and under	0.049 (0.07)
6 to 17	0.045 (0.25)
18 to 35	0.023 (0.35)
36 to 59	0.002 (0.07)
60 to 74	**0.097*** (4.38)**
75 to 84	**0.112*** (4.96)**
85 and older	**0.377*** (13.35)**
NA	-0.081 (-0.50)

Supplementary Table S5: Changes in the age categories before and during the COVID-19 pandemic. Note: Bivariate Poisson regressions between pandemic dummy variable and monthly numbers of admissions in different age categories (absolute numbers). Pandemic dummy is coded as 1 if month is from March 2020 to April 2023, 0 before March 2020. The two months after the pandemic (May and June 2023) are set to missing. Missing values are marked as NA (not available). Significant results in bold; *** p ≤ 0.001, ** p ≤ 0.01, * p ≤ 0.05. Constant terms not shown for clarity.

Age Category	Status in COVID-19 confirmed	Fold Change	P-value
5 and under	**under-represented*****	0.134	< 0.001
6 to 17	under-represented	0.599	0.197
18 to 35	**under-represented****	0.588	0.001
36 to 59	**under-represented*****	0.702	< 0.001
60 to 74	**under-represented*****	0.765	< 0.001
75 to 84	**over-represented*****	1.172	< 0.001
85 and older	**over-represented*****	1.587	< 0.001
NA	**over-represented****	2.106	0.004

Supplementary Table S6: Number of admissions in different age categories for corpses with confirmed SARS-CoV-2 infection compared to all admissions. Note: Parameters to calculate the p-value for under- or over-representation are based on the cumulative distribution function of the hypergeometric distribution. Fold change as compared to expectations based on all data during the observation period. Missing values are marked as NA (not available). Significant results in bold; *** p ≤ 0.001, ** p ≤ 0.01.
